# Causes and Cure of Diseases of the Feet, with Practical Suggestions as to Their Clothing

**Published:** 1862-11

**Authors:** C. H. Cleveland


*Causes and Cure of Diseases of the Feet, with practical suggestions as to their clothing.* By C. H. Cleveland, M. D.—*Illustrated.*

This is a very concise and comprehensive work upon the feet. It treats of them both in regard to health and disease; the methods of keeping them in good condition are fully detailed. The diseases to which they are liable, and the treatment of these affections, are clearly and concisely considered.

This is a work that is interesting to everybody, for almost all have abused or neglected their feet. Many are content to give them no attention, so long as they can toddle round on them. After a while come the aches and pains, and oh, what pains: then they begin to ask, what shall I do to save my feet? oh, these corns; oh, these pinched toes—what aches and throes are made by neglect and shoes; shoes too short, too tight, too narrow, with feet in them all cramped, ambling through the world, evidently belonging to that class of persons who get their punishment as they go along.

If the suggestions made in the work be followed, all these difficulties will be obviated, or if they already exist, be remedied. In this work is brought together, arranged and systematized, a large amount of matter on this subject, that is scattered through medical literature, in such a manner that it is of no avail to the people, and by no means in the best shape for the physician; but in this work it is brought together with much that is new and valuable, so as to be most useful to the physician and the people. It is a work that should be in every family in the country.

